# Supplementary material for: Methuosis Contributes to Jaspine-B-Induced Cell Death
Source: Int J Mol Sci. 2022 Jun 29;23(13):7257. doi: 10.3390/ijms23137257 (PMC9267113; doi:10.3390/ijms23137257)
Supplement: Supplementary file 1 [file ijms-23-07257-s001.zip › ijms-1779340-supplementary.pdf]

Supplementary Materials

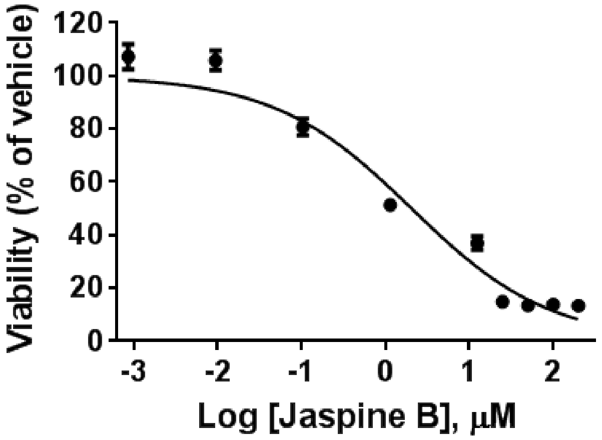

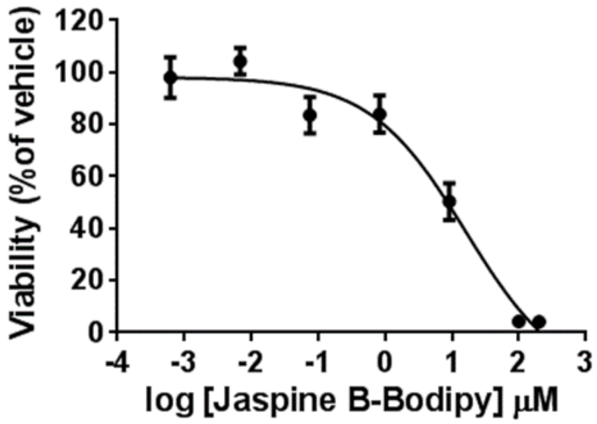

Figure S1. A549 cells were treated with different concentrations of JB (A) or Jaspine B-Bodipy (B) for 24 h and cell viability was assessed with 3-(4,5-dimethylthiazol- 2-yl)-2,5-diphenyl tetrazolium bromide (MTT). Results are the mean ± SD of three experiments performed in triplicate and are expressed as the percentage of the viability compared to the control. Data analysis using the Log(inhibitor) vs. response - variable slope (four parameters) equation (GraphPad Prism) afforded a CC50 of 2.05 μM for JB and of 16 μM for Jaspine B-Bodipy.

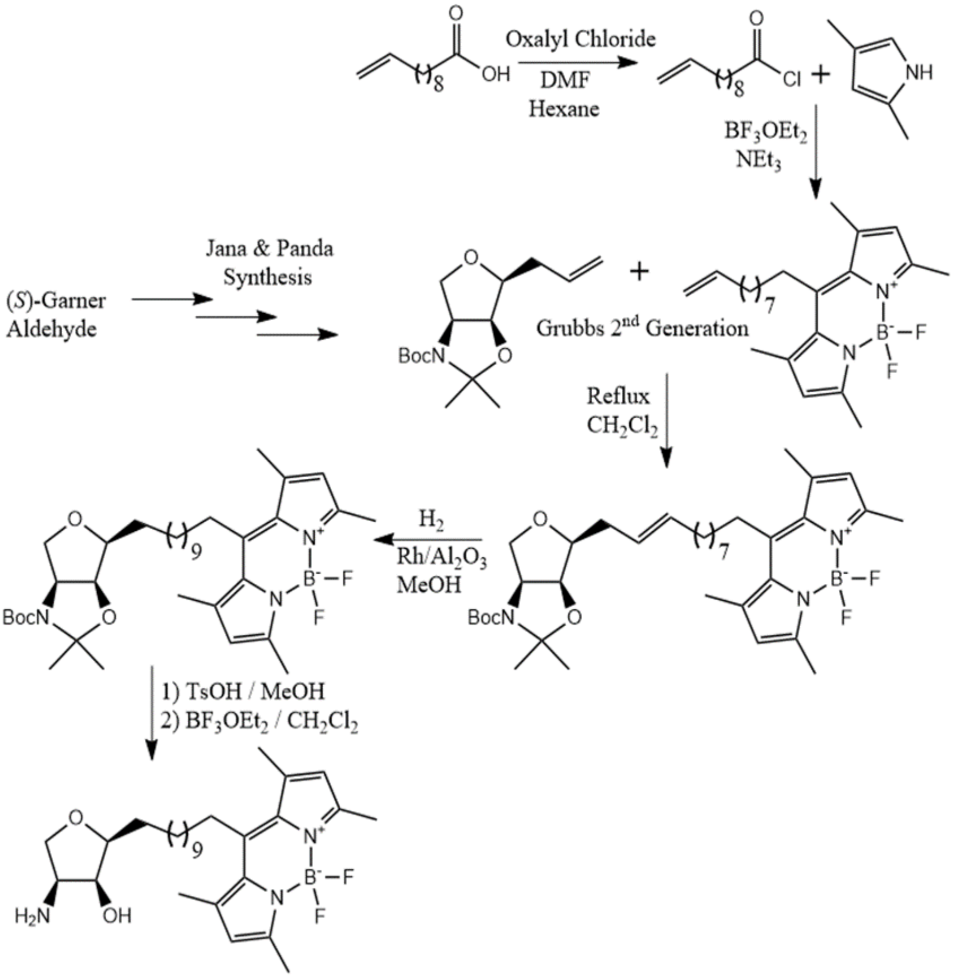

Figure S2. Chemical synthesis of Jaspine B-Bodipy. BF3OEt2, boron trifluoride diethyl etherate; DMF, dimethyl formamide; NEt3, Triethylamine; TsOH, p-toluenesulfonic acid. Jana, A.K.; Panda, G. Ref. 1: Stereoselective synthesis of Jaspine B and its C2 epimer from Garner aldehyde. RSC Adv.**2013**, *3*, 16795–16801, doi:10.1039/c3ra41778f.

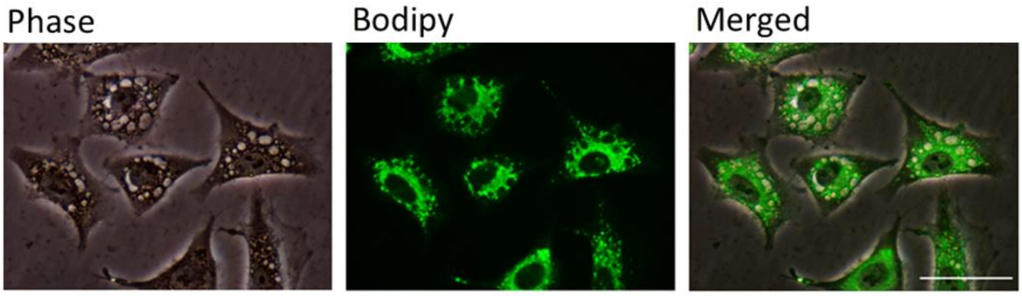

Figure S3. Induction of cell vacuolization by JB-Bodipy. A549 cells treated with 30 μM JB-Bodipy for 2 h and were visualized by Fluorescence microscopy. Scale bar: 50 μm. Images are representative of six separate experiments.

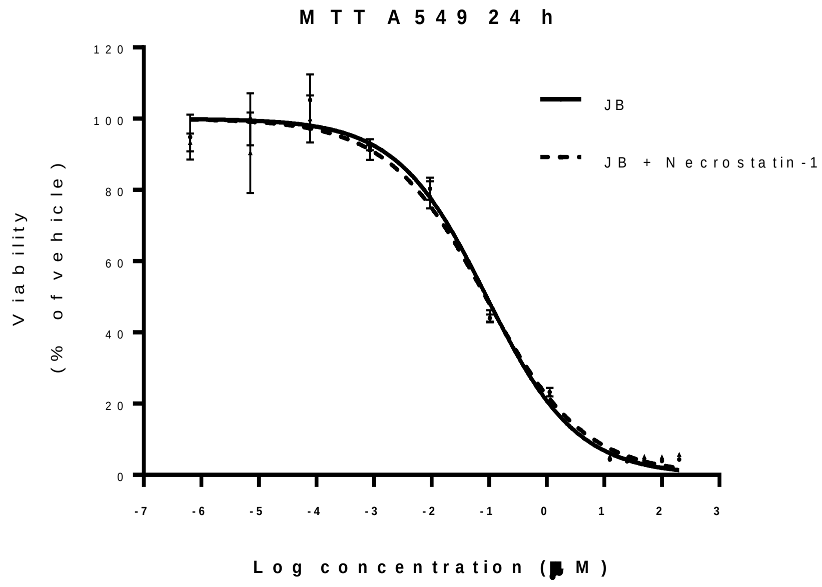

Figure S4. Jaspine B (JB) does not cause cell death by necrosis in A549 cells. A549 cells were treated with different concentrations of JB for 24 h after a 1-hour pre-incubation of 50 µM Necrostatin-1. Results are the mean ± SD of two experiments in triplicate ($P > 0.05$).

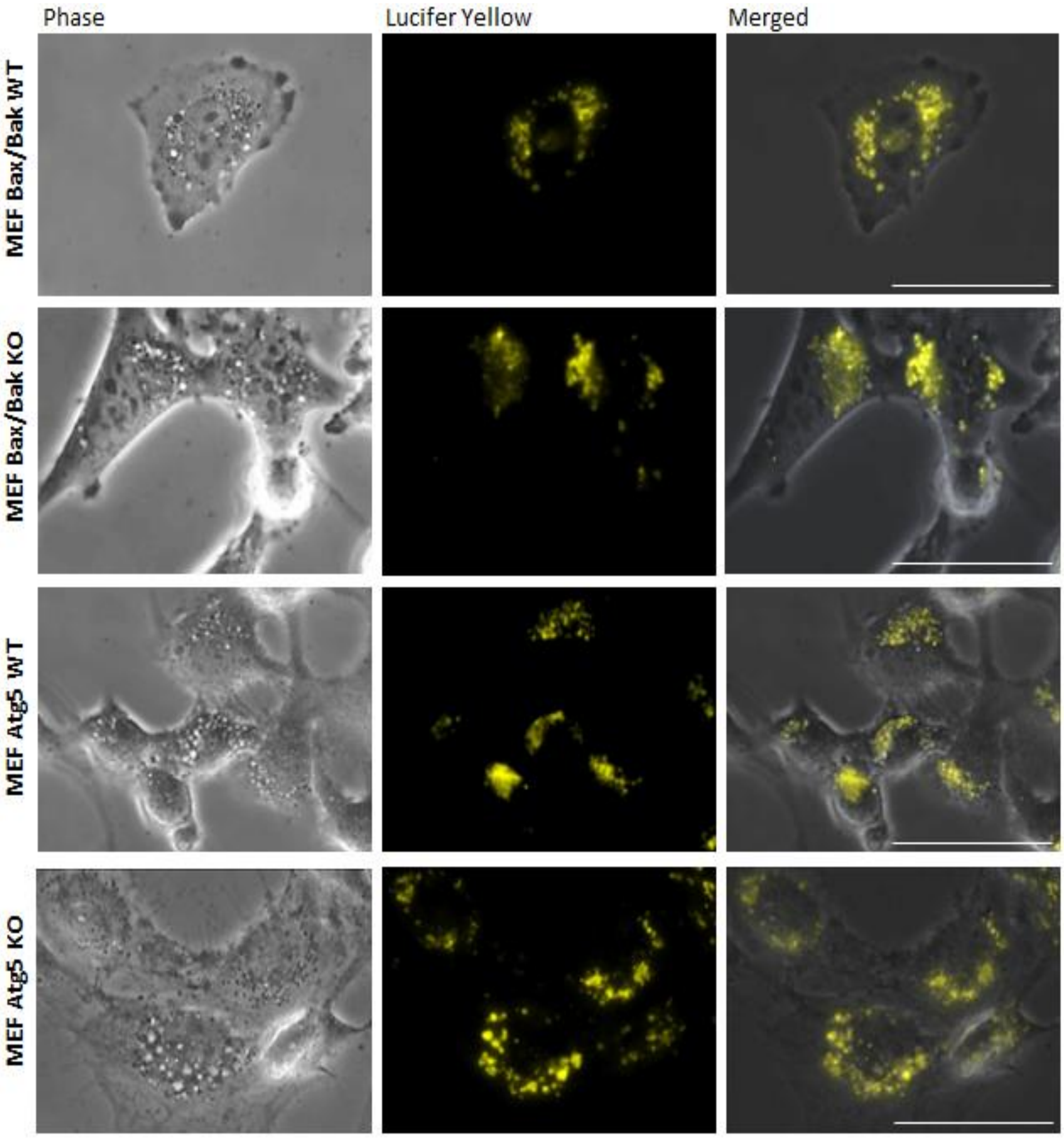

Figure S5. Vacuoles induced by Jaspine B (JB) in different murine cell lines are originated by macropinocytosis. Bcl-2-associated X protein-/-/Bcl-2-antagonist/killer 1-/- (BAX/BAK KO), autophagy related gene 5-/- (Atg5 KO) and Atg5+/+ (Atg5 WT) mouse embryonic fibroblasts (MEF) were treated with 5 μM JB and 0.5 mg/ml of Lucifer yellow (LY) and incorporation of LY in the vacuoles induced by JB was evaluated 4 h later by phase microscopy. Images are representative of two experiments. Scale bar: 50 μm.
